# Tetartohedral twinning could happen to you too

**DOI:** 10.1107/S0907444912006737

**Published:** 2012-03-16

**Authors:** Pietro Roversi, Eric Blanc, Steven Johnson, Susan Mary Lea

**Affiliations:** a Sir William Dunn School of Pathology, Oxford University, South Parks Road, Oxford OX1 3RE, England; b King’s College Centre for Bioinformatics (KCBI), School of Physical Sciences and Engineering, King’s College London, Strand, London WC2R 2LS, England; c MRC Centre for Developmental Neurobiology, New Hunt’s House, King’s College London, Guy’s Campus, London SE1 1UL, England

**Keywords:** tetartohedral twinning

## Abstract

Tetartohedral crystal twinning is discussed as a particular case of (pseudo)merohedral twinning when the number of twinned domains is four. Tetartohedrally twinned crystals often possess pseudosymmetry, with the rotational part of the pseudo­symmetry operators coinciding with the twinning operators. Tetartohedrally twinned structures from the literature are reviewed and the recent structure determination of tetartohedrally twinned triclinic crystals of human complement factor I is discussed.

## Abstract 2

A review of published tetartohedrally twinned macromolecular structures is presented, together with details of the recent structure determination of triclinic tetartohedrally twinned crystals of human complement factor I.

## 1. Introduction

When crystal (pseudo)merohedral twinning arises from four twinned crystal domains, the twinning is called tetartohedral. In this manuscript, we review published tetartohedrally twinned macromolecular structures (see Table 1 ▶; Rosendal *et al.*, 2004 ▶; Barends *et al.*, 2005 ▶; Gayathri *et al.*, 2007 ▶; Fernández-Millán *et al.*, 2008 ▶; Yu *et al.*, 2009 ▶; Anand *et al.*, 2007 ▶; Leung *et al.*, 2011 ▶) and find that this type of twinning is almost always accompanied by pseudosymmetry, with the twinning operators coinciding with the rotational parts of the pseudosymmetry. To discuss a few of the issues that arise when working with tetartohedrally twinned crystals, we also illustrate the determination of the structure of tetartohedrally twinned triclinic crystals of human complement factor I (Roversi *et al.*, 2011 ▶).

## 2. Tetartohedral twinning is (pseudo)merohedral twinning with *N*_twins_ = 4

If the crystalline sample exposed to X-rays is made of *N*_twins_ single crystals, the twinning is described in terms of the relative sizes of the *N*_twins_ domains (the twin fractions α*_k_*) and the set of matrices **T** *_k_* that represent the twinning operators. When the twinning operators leave the crystal lattice (almost) unchanged, the twinning is called merohedral (pseudomero­hedral). The X-ray diffraction spots from all of the twinned domains (almost) overlap and the diffracted intensity can be written
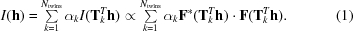
As already mentioned, when *N*_twins_ = 4 the structure is said to be tetartohedrally twinned. As for any type of (pseudo)­merohedral twinning, detection of tetartohedral twinning is possible at an early stage, after a set of diffraction intensities has been collected, by performing a number of tests that analyse the crystal intensity statistics (see Yeates, 1997 ▶)^1^. The formulae needed to estimate twin fractions from tetartohedrally twinned data have been described in Yeates & Yu (2008 ▶).

If the extent of twinning is small and/or is obscured by the presence of noncrystallographic symmetry, and especially when the NCS axes coincide with the directions of twinning (the latter introducing deviations from the intensity statistics used to derive the twinning tests), it can also be the case that twinning can only be confirmed at a stage as late as that of refinement of the model. Fortunately, in this case the availability of the model allows statistical tests on the calculated intensities, which can help in estimation of the twin fractions: intensity statistics in the presence of NCS and twinning have been discussed and illustrated in Lebedev *et al.* (2006 ▶) and Zwart *et al.* (2008 ▶).

For a discussion of experimental phasing see Dauter (2003 ▶) and for a discussion of molecular replacement in the presence of crystal twinning see Redinbo & Yeates (1993 ▶), Breyer *et al.* (1999 ▶) and Jameson *et al.* (2002 ▶). Generally speaking, whenever data sets from several different twinned samples have been measured and estimates of the twinning fractions have been obtained for each sample, if possible one should avoid working with data sets from crystals for which all the twinned fractions are close to 1/*N*_twins_ (‘perfect twinning’). Of course, the closer the sample is to perfect twinning, the greater the need for the accurate estimation of twinning fractions based on *I*_**h**_^calc^ from each twin domain, which is only possible if the structure is available. This in turn means that only towards the end of the structure-determination process will the details of each twinned sample be properly understood and the optimal choice of sample/data set be possible.

## 3. Structural refinement against tetartohedrally twinned data

Various strategies are possible when refining against twinned data and tetartohedral twinning is not an exception. The simplest approach would involve detwinning the experimental intensities on the basis of the current estimates for the twin ratios by using the current model and *I*_**h**_^calc^ from each twin domain. Structural refinement can then be carried out against these intensities, leading to a new model and a new round of estimation of twin ratios and so on, hopefully to convergence (see, for example, the refinement of PDB entry 3eop; Yu *et al.*, 2009 ▶). This strategy may suffer from instability and its convergence may be slow.

In a second approach, the refinement target function can be defined taking twinning into account and refinement carried out against the twinned intensities. Ideally, refinement of the twinning ratios should be carried out at the same time as the refinement of the structural parameters (scale factors, atomic coordinates and *B* factors, occupancies *etc.*), possibly including joint second derivatives of the refinement target function with respect to twin fractions and other parameters. The least-squares refinement program *SHELXL*-97 has long allowed joint structural refinement against tetartohedrally twinned diffraction intensities (Herbst-Irmer & Sheldrick, 1998 ▶). It refines all parameters in the same conjugate-gradient or matrix-inversion run. If the matrix of the second derivatives of the target function with respect to the parameters is inverted, it is possible to obtain the correlations between the twin fractions and the other parameters of the model and error estimates of the twin fractions.

To make refinement computationally simpler, the twin fractions can be optimized while holding the other parameters fixed and *vice versa*, alternating cycles of refinement of twin fractions and structural parameters. A protocol to perform refinement of the model against tetartohedrally twinned intensities was included in the supplementary information of Barends *et al.* (2005 ▶). This protocol makes use of the program *CNS* and it relies on initial estimation of the twin fractions, which are subsequently kept fixed during the least-squares structural refinement. More recently, the refinement program *REFMAC*5 enabled the initial detection of tetartohedral twin operators, initial estimation of the twin fractions and their maximum-likelihood optimization in between cycles of refinement of structural parameters (Murshudov *et al.*, 2011 ▶).

Of course, as is the case with all refinements against intensities from merohedrally twinned crystals and/or crystals that possess NCS, special care should be taken in assigning free *R* flags so that NCS-related and/or twin-related reflections either belong to the free or to the working set, *i.e.* NCS/twin-related reflections should not be distributed across the two sets (Kleywegt & Brünger, 1996 ▶). *REFMAC*5 internally changes free *R* flags so that twin-related reflections belong to the either the free or the working set.

## 4. Tetartohedrally twinned structures in the literature

Keyword searches in the Protein Data Bank and the literature (*via* the PubMed server) returned a number of published crystal structures from tetartohedrally twinned crystals.^2^ We summarize them in Table 1 ▶.

In many of these structures the noncrystallographic symmetry operators are close to true crystallographic symmetry (pseudosymmetry; Zwart *et al.*, 2008 ▶; Appendix *A* [App appa]) and the group of the NCS rotations coincides with that of the twinning operators. Interestingly, most of these structures are trigonal, with the merohedral twinning operators and the NCS belonging to point group 222; the twofold axes are aligned along *a*, a* and *c* so as to create apparent 622 point symmetry. One structure (PDB entry 2xrc; see below) is pseudomero­hedrally twinned, triclinic *P*1, but with a pseudo-orthorhombic cell and the NCS and the twinning twofolds also aligned with crystal axes. The only published tetartohedrally twinned structure for which the group of the NCS rotations and one of the twinning operators do not coincide is PDB entry 3eop, where the twofold NCS operator and the crystal symmetry together have 321 symmetry, while the twinning has 222 symmetry (the two groups sharing only the twofold along *a*).

## 5. Tetartohedrally twinned crystals of human complement factor I

The crystal structure of human complement factor I (fI) was described in Roversi *et al.* (2011 ▶). The crystals were triclinic and tetartohedrally twinned. In this manuscript, we examine the analysis of the crystal symmetry, the detection of the tetartohedral twinning and the protocol followed for initial phasing, model building and refinement of the structure against the tetartohedrally twinned diffraction data.

The fI crystals appeared to be frayed at the ends, which may indicate several crystalline layers stacking to form each sample, but otherwise had sharp edges, could be grown reproducibly and gave diffraction patterns that could be successfully indexed by invoking a single lattice (Roversi *et al.*, 2011 ▶).

Several samples were exposed to X-­rays and diffraction data sets were measured, the best diffracting of which (2.4 Å resolution) was collected in September 2009 at 100 K using X-­rays of wavelength 0.97630 Å on beamline I03 at the Diamond Light Source, Harwell, England. The data were originally indexed and scaled in a primitive orthorhombic 222 lattice with the unit-cell parameters reported in Table 2 ▶. Analysis with *POINTLESS* and *phenix.xtriage* (Zwart *et al.*, 2008 ▶) suggested a primitive 222 lattice and space group *P*2_1_2_1_2.

No problems were initially noticed, apart from the fact that the cumulative intensity distribution (not shown), other overall intensity statistics and the results of the *L*-test (see Table 3 ▶) departed from what would be expected from good to reasonable untwinned data. As there are no (pseudo)mero­hedral twin laws possible for these orthorhombic crystals, *phenix.xtriage* concluded that

there could be a number of reasons for the departure of the intensity statistics from normality. Overmerging pseudo-symmetric or twinned data, intensity-to-amplitude conversion problems as well as bad data quality might be possible reasons. It could be worthwhile considering reprocessing the data.

Had one attempted scaling in a lower symmetry space group, the scaling statistics would have shown only a marginal improvement upon lowering of the symmetry (see Table 2 ▶). In agreement with the these scaling statistics, the κ = 180° section of the self-rotation function for this crystal shows almost perfect 222 symmetry, with three peaks at 94, 93 and 92% of the origin and along the directions (ω = 89.9°, ϕ = 0.0°), (ω = 89.9°, ϕ = 89.9°) and (ω = 0.0°, ϕ = 0.0°), respectively. Retrospectively, once the structure was solved in *P*1 and the tetartohedral twin fractions were calculated with *REFMAC*5 it appeared that this crystal (like all other fI triclinic crystals measured but one) was almost perfectly tetartohedrally twinned, *i.e.* the four twin fractions were all close to 1/4 (see Table 6, last column), a special case of the condition α_*k*_ + α_*k*′_ = 1/2 that makes twinned crystals most problematic (‘perfect twin’; Yeates, 1997 ▶).

Further clues to the fact that the crystals were not ortho­rhombic came from molecular-replacement efforts in *P*2_1_2_1_2 using *Phaser* and searching with several models of domains homologous to the serine protease domain (43% of the structure). The searches consistently yielded a pair of placements (with very equivalent scores) which shared the rotation-function maximum but differed by a shift of almost 6 Å along *c* in the translation-function maximum. This could be interpreted as an indication of lower symmetry, but the observation was originally ignored and model building attempted starting from the top-scoring placement in *P*2_1_2_1_2, without much success.

**Figure 1:**
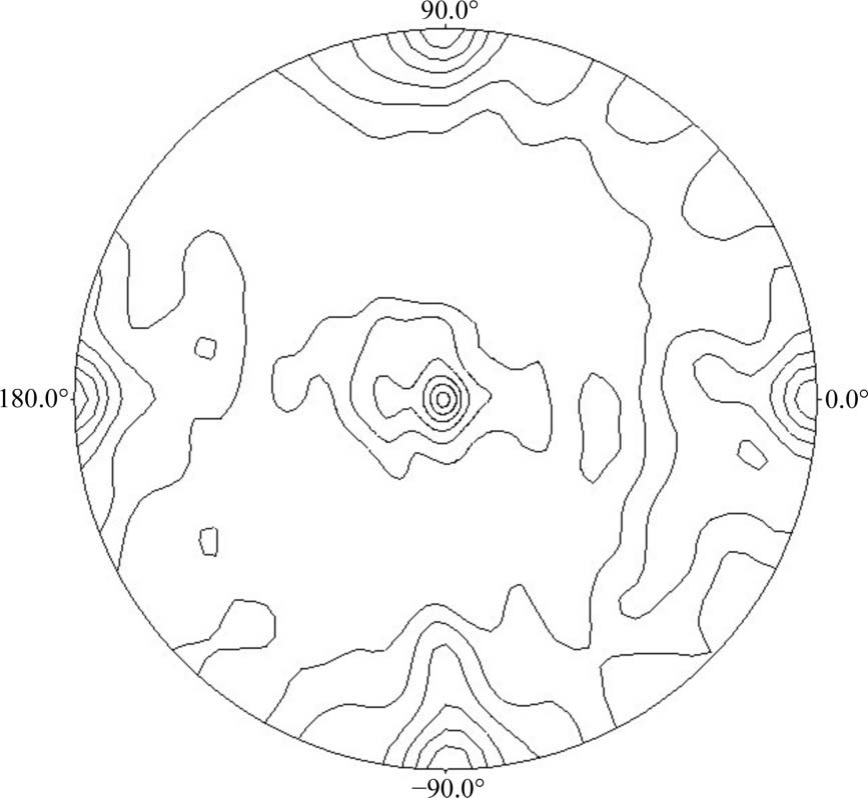
Triclinic human complement factor I: κ = 180° section of the self-rotation function in the resolution interval 52–2.7 Å. Contour levels: 1–6σ in steps of 1σ. Three peaks are visible at ω = 90.7°, ϕ = 90.6°, κ = 180° (6.6σ), ω = 89.7°, ϕ = 0.0°, κ = 180° (6.6σ) and ω = 0°, ϕ = 0°, κ = 180° (6.2σ). Computed with the program *POLARRFN*.

In November 2009, an fI crystal gave a 2.70 Å resolution diffraction data set on beamline I02 at the Diamond Light Source, on analysis of which *phenix.xtriage* (Zwart *et al.*, 2008 ▶) indicated the need to lower the symmetry to *P*2_1_ with the monoclinic axis along the longest dimension and a β angle of 90.2°. The scaling statistics also agreed with the data merging better as monoclinic (see Table 4 ▶). The 222 symmetry in the κ = 180° section of the self-rotation function computed from these data in *P*1 is still apparent in Fig. 1 ▶, but the self-rotation function maxima are only 80% of the origin and along directions ω = 90.7°, ϕ = 90.6°, ω = 89.7°, ϕ = 0.0° and ω = 0.0°, ϕ = 0.0° and thus are neither as intense nor as orthogonal to each other as would be expected for ortho­rhombic crystals.

Indeed, reprocessing the data in *P*2_1_ improved the value of *R*_meas_ a.k.a. *R*_r.i.m._ (see Table 4 ▶). This assumed two molecules per asymmetric unit and pseudomerohedral twinning with operators *h*, *k*, *l* and −*h*, −*k*, *l*, with the two twin fractions estimated at around 0.49–0.5. However, the intensity statistics still indicated problems with the data (see Table 5 ▶). After a few more unsuccessful attempts at refining the structure in *P*2_1_, the symmetry was eventually lowered to *P*1, invoking four molecules in the asymmetric unit and tetartohedral twinning along the crystal axes (operators *h*, *k*, *l*; −*h*, −*k*, *l*; *h*, −*k*, −*l*; −*h*, *k*, −*l*).

**Figure 2:**
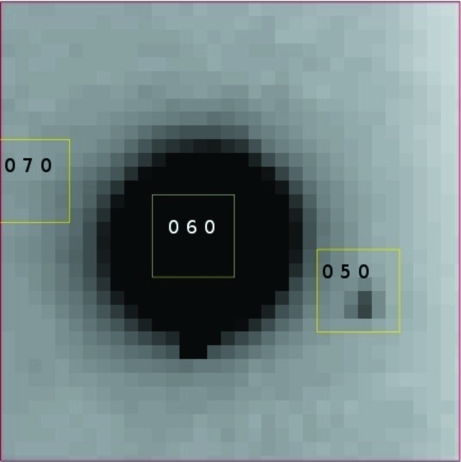
Triclinic human complement factor I: detail of one of the diffraction frames collected on beamline I02 at Diamond in November 2009. Indexing and prediction was performed in *MOSFLM*. The yellow boxes show the predicted location of the spots, with their *hkl* indices in black. The reflection 050 appears next to the much stronger 060. After integration, *I*_050_/σ(*I*_050_) = 294/24, *i.e.* the intensity of the reflection is weak but still ten times its σ.

In keeping with triclinic symmetry and pointing to the fact that the crystals are not monoclinic *P*2_1_, the reflection 050 had nonzero intensity in more than one data set (Fig. 2 ▶ shows one such measurement). Although violations of the systematic absences of higher symmetry space groups can be explained for example by multiple scattering (Renninger, 1937 ▶) and/or anisotropy of anomalous scattering (Templeton & Templeton, 1980 ▶), in the context provided by the merging and intensity statistics, self-rotation function and molecular-replacement hits, the repeated measurements of such a reflection from more than one crystal sample were taken as additional evidence that the fI crystals were indeed triclinic.

The structure of the triclinic human complement fI crystals was eventually determined by sequential molecular replacement in *P*1, searching against the tetartohedrally twinned intensities with *Phaser* and search models from homologous individual domains. The initial solution was followed by iterative model building in *Coot* (Emsley *et al.*, 2010 ▶) and refinement in *REFMAC*5 (Roversi *et al.*, 2011 ▶). The four copies of the molecule in the cell are arranged in a pseudo­orthorhombic packing which almost follows *P*2_1_2_1_2 symmetry except that the two molecules related by the twofold axis along *c* are also shifted with respect to each other by about 6 Å along the same direction.

Tight NCS restraints were initially used and gradually released during the course of model building and refinement: whenever the current electron density showed surface loops and crystal contacts that differed in the four copies of the molecule these regions were omitted from the part of the structure that was NCS-restrained. In the final model, approximately 35% of the structure had to be excluded from the NCS restraints.

Refinement statistics are reported in Roversi *et al.* (2011 ▶). The *REFMAC*5 estimates of the twinning fractions at the end of the refinement and building process are reported in Table 6 ▶. Table 6 ▶ also reports the *R* ^obs^_twin_ and *R* ^calc^_twin_ values (Lebedev *et al.*, 2006 ▶; for the use of statistical agreement indicators on observed and calculated intensities in order to investigate twinning and NCS, see also Lee *et al.*, 2003 ▶). As expected, *R* ^obs^_twin_ < *R* ^calc^_twin_ for all twinning operators, placing the factor I crystals in the regions of the RvR plot that is characteristic of twinned crystals with rotational pseudosymmetry (RPS; Lebedev *et al.*, 2006 ▶). 

## 6. Conclusions

The relatively recent occurrence in the literature of several cases of tetartohedrally twinned structures suggests that this form of twinning is not as infrequent as one might wish it to be (with the additional possibility that further tetartohedrally twinned structures may be lurking in the PDB, having been determined and deposited with the twinning going un­detected). Tetartohedral twinning could happen to you too!

Fortunately, careful analysis of intensity statistics and rotational symmetry can help to overcome the difficulties associated with this type of twinning, even in the presence of the potentially confusing shared rotational NCS and twinning symmetry. In addition, excellent statistical tools are now available in a number of data-processing/analysis programs, *e.g. phenix.xtriage*, to detect potential twinning laws and guide the crystallographer towards the correct symmetry, twinning laws and twinning fraction. Once detected and characterized, tetartohedral twinning is also relatively simple to handle thanks to a number of good macromolecular refinement programs, notably *CNS*, the least-squares program *SHELXL*-97 and the latest version of the maximum-likelihood refinement program *REFMAC*5. Tetartohedral twinning is not a fatal disease. Only, to quote Petrus Zwart

By now you should be a crystallographic hypochondriac

(Zwart, 2009 ▶).

## 

## Figures and Tables

**Table 1:** Summary of tetartohedrally twinned structures in the literature

PDB code	Apparent symmetry	True symmetry	*G*_twin_	*G*_NCS_	*R*	*R*_free_	Twin operators†
1qzw‡	*P*6_4_22	*P*3_1_	222	222	0.340	0.387	i
2pi8§	*P*6_4_22	*P*3_1_	222	222	0.200	0.238	i
2h6r¶	*P*6_4_22	*P*3_1_	222	222	0.213	0.278	i
3eop††	*P*6_4_22	*P*3_1_	222	2	0.182	0.238	i
2y9a, 2y9b, 2y9c, 2y9d‡‡	*P*6_1_22	*P*3_1_	222	222	0.277	0.321	i
3nuz§§	*P*6_5_22	*P*3_2_	222	222	0.153	0.182	i
2pk2¶¶	*I*23	*H*3	222	222	0.272	0.306	ii
2krc†††	*P*2_1_2_1_2	*P*1	222	222	0.200	0.240	iii

† (i) *h*, *k*, *l*; −*k*, −*h*, −*l*; −*h*, −*k*, *l*; *k*, *h* −*l*; (ii) *h*, *k*, *l*; −*h*/3, *k*/3, 4*l*/3; *h*/3, −*k*/3, −4*l*/3; −2*h*/3, −*k*/3, −4*l*/3; (iii) *h*, *k*, *l*; −*h*, *k*, −*l*; −*h*, −*k*, *l*; *h*, −*k*, *l*.

‡ Rosendal *et al.* (2004 ▶).

§ Barends *et al.* (2005 ▶).

¶ Gayathri *et al.* (2007 ▶).

†† Yu *et al.* (2009 ▶).

‡‡ Leung *et al.* (2011 ▶).

§§ Joint Center for Structural Genomics, unpublished work.

¶¶ Anand *et al.* (2007 ▶).

††† Roversi *et al.* (2011 ▶).

**Table 2:** Summary of the September 2009 X-ray diffraction data quality for human complement factor I (PDB entry 2xrc) integrated and scaled in three different space groups For the present manuscript, all data processing was repeated with the *xia*2 suite of programs (Winter, 2010 ▶) running *XDS* (Kabsch, 2010*a* ▶,*b* ▶) for indexing and integration and *SCALA* (Evans, 2006 ▶) for scaling.

Symmetry (*Z*)	*P*1 (4)	*P*2_1_ (4)	*P*2_1_2_1_2 (4)
Unit-cell dimensions (Å, °)	*a* = 72.02, *b* = 235.92, *c* = 40.47		
Unit-cell angles (°)	α = 89.97, β = 90.24, γ = 90.01	α = γ = 90, β = 90.24	α = β = γ = 90
Resolution (Å)	78.55–2.42 (2.48–2.42)		
*R*_merge_	0.06 (0.38)	0.08 (0.38)	0.10 (0.44)
*R*_meas_ a.k.a. *R*_r.i.m._	0.07 (0.51)	0.09 (0.47)	0.11 (0.50)
Unique observations	89922 (4462)	50118 (6616)	27178 (3788)
〈*I*/σ(*I*)〉	11.2 (2.0)	12.1 (2.7)	13.4 (3.2)
Completeness	0.89 (0.60)	0.98 (0.89)	0.99 (0.94)
Multiplicity	2.2 (1.9)	3.9 (2.8)	7.3 (4.8)

**Table 3:** Summary of intensity statistics for the September 2009 complement factor I triclinic data The statistics were computed using *phenix.xtriage* with data between 10 Å and a maximum resolution chosen such that the data with *I*/σ(*I*) > 3.00 still give 85% completeness. Expected intensity statistics for untwinned and perfectly twinned crystals were taken from Yu *et al.* (2009 ▶) and Stanley (1955 ▶).

Symmetry (*Z*)	*P*1 (4)	*P*2_1_ (4)	*P*2_1_2_1_2 (4)	No twin	Perfect twin
Resolution range (Å)	10–2.43	10–2.87	10–2.77		
〈*I*^2^〉/〈*I*〉^2^, acentric (centric)	1.748	1.745 (2.282)	1.666 (2.436)	2.0 (3.0)	1.5 (2.0)
〈*F*^2^〉/〈*F*〉^2^, acentric (centric)	0.842	0.843 (0.740)	0.856 (0.719)	0.785 (0.637)	0.885 (0.785)
〈|*E*^2^ − 1|〉, centric (acentric)	0.642	0.631 (0.841)	0.602 (0.896)	0.736 (0.968)	0.541 (0.736)
|*L*| (acentric)	0.424	0.418	0.403	0.500	0.375
〈*L*^2^〉 (acentric)	0.250	0.244	0.228	0.333	0.200
Multivariate *Z* score *L*-test	5.8	6.8	8.1	<3.5	>3.5

**Table 4:** Summary of the November 2009 X-ray diffraction data quality for human complement factor I (PDB entry 2xrc) integrated and scaled in three different space groups For the present manuscript, all data processing was repeated with the *xia*2 suite of programs (Winter, 2010 ▶) running *XDS* (Kabsch, 2010*a* ▶,*b* ▶) for indexing and integration and *SCALA* (Evans, 2006 ▶) for scaling.

Symmetry (*Z*)	*P*1 (4)	*P*2_1_ (4)	*P*2_1_2_1_2 (4)
Unit-cell dimensions (Å)	*a* = 71.32, *b* = 234.72, *c* = 40.30		
Unit-cell angles (°)	α = 89.98, β = 90.18, γ = 90.03	α = γ = 90, β = 90.18	α = β = γ = 90
Resolution (Å)	53–2.70 (2.77–2.70)		
*R*_merge_	0.08 (0.35)	0.09 (0.39)	0.22 (0.80)
*R*_meas_ a.k.a. *R*_r.i.m._	0.09 (0.42)	0.10 (0.44)	0.23 (0.84)
Unique observations	64339 (4384)	35959 (2438)	19491 (1355)
〈*I*/σ(*I*)〉	10.1 (2.4)	12.7 (3.2)	9.5 (3.3)
Completeness	0.90 (0.84)	0.99 (0.94)	1.00 (0.99)
Multiplicity	2.8 (2.5)	5.1 (4.3)	13.1 (10.5)

**Table 5:** Summary of intensity statistics for the November 2009 complement factor I triclinic data The statistics were computed using *phenix.xtriage* with data between 10 Å and a maximum resolution chosen such as the data with *I*/σ(*I*) > 3.00 still give 85% completeness. Expected intensity statistics for untwinned and perfectly twinned crystals were taken from Yu *et al.* (2009 ▶) and Stanley (1955 ▶).

Symmetry (*Z*)	*P*1 (4)	*P*2_1_ (4)	*P*2_1_2_1_2 (4)	No twin	Perfect twin
Resolution range (Å)	10–3.35	10–3.16	10–3.10		
〈*I*^2^〉/〈*I*〉^2^, acentric (centric)	1.779	1.756 (2.067)	1.632 (2.476)	2.0 (3.0)	1.5 (2.0)
〈*F*^2^〉/〈*F*〉^2^, acentric (centric)	0.842	0.846 (0.784)	0.869 (0.732)	0.785 (0.637)	0.885 (0.785)
〈|*E*^2^ − 1|〉, centric (acentric)	0.638	0.632 (0.750)	0.579 (0.928)	0.736 (0.968)	0.541 (0.736)
|*L*| (acentric)	0.420	0.417	0.380	0.500	0.375
〈*L*^2^〉 (acentric)	0.244	0.240	0.204	0.333	0.2
Multivariate *Z* score *L*-test	5.9	5.9	11.1	<3.5	>3.5

**Table 6:** Human fI: estimation of agreement statistics and twinning fractions The *R* ^obs^_twin_, *R* ^calc^_twin_, Britton α, *H* 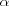 and ML α statistics were computed with *phenix.xtriage* using the same subset of data as in Table 5 ▶. *R* ^obs^_twin_ and *R* ^calc^_twin_ are the statistics introduced in Lebedev *et al.* (2006 ▶). The last two columns report the *REFMAC*5-estimated twin fractions when refining the final model against the 2.7 Å November 2009 data set and the 2.4 Å September 2009 data set, respectively.

*P*1 twin operator	*R*^obs^_twin_	*R*^calc^_twin_	Britton α	*H* α	ML α	*REFMAC*5 α (Nov. 2009)	*REFMAC*5 α (Sept. 2009)
*h, k, l*	—	—	—	—	—	0.33	0.28
−*h*, −*k, l*	0.169	0.436	0.331	0.337	0.249	0.21	0.22
*h*, −*k*, −*l*	0.172	0.438	0.316	0.326	0.276	0.10	0.22
−*h, k*, −*l*	0.086	0.336	0.420	0.419	0.370	0.36	0.28

## Footnotes

1 Of course, twinning can be also thought of as a very special case of powder diffraction, intensity statistics for which are discussed in Bricogne (1991 ▶).

2 The structure of MJ0729, a CBS-domain protein from *Methanococcus jannaschii*, was reported to suffer from tetartohedral twinning (Fernández-Millán *et al.*, 2008 ▶), but it has not been fully refined nor deposited at the time of this writing and was therefore not included in Table 1 ▶.

## Appendix A

### A1. Twinning operators coinciding with the rotational part of pseudosymmetry

A survey of the tetartohedrally twinned structures that have appeared to date in the literature suggested that in most cases the tetartohedral twinning operators are aligned with the rotational parts of the noncrystallographic symmetry operators and that the latter in turn are close to true crystallo­graphic symmetry, a situation known as pseudosymmetry (Zwart *et al.*, 2008 ▶). In this Appendix, we derive a formula that illustrates the contributions to the diffracted intensity from the part of the structure that follows the pseudosymmetry and the part that does not and their interplay with the twinning fractions.

Let us write the electron density in the asymmetric unit of the crystal as

where ρ^NCS^(**x**) is the part of density in the asymmetric unit that follows noncrystallographic symmetry (NCS), *i.e.* electron density from a reference set of atoms and its NCS-related copies, and ρ^noNCS^(**x**) is the electron density for the remaining part of the asymmetric unit, which cannot be described using a reference copy and NCS operators. The portion of the electron density that obeys the NCS can be written using the *J* NCS operators starting from the electron density for the reference copy, labelled ρ_1_(**x**),

where **R**_*j*_ is the rotation matrix and **t** *_j_* is the translation vector of the *j*th noncrystallographic symmetry operator.

The structure factor is the Fourier transform of the unit-cell electron density ρ_cell_(**x**); following the above notation,

where the *i*th crystallographic operator **G** *_i_* in the crystal space group *G* acts as

In other words, **S**_*i*_ is the rotation matrix and **t**_*i*_ is the translation vector of the *i*th space-group symmetry operator. Λ is the set of crystal lattice translations.

As we saw earlier, when the crystals are (pseudo)mero­hedrally twinned evaluation of the diffraction intensity (1) involves the calculation of the structure factors evaluated at reciprocal-lattice vectors rotated by the twinning operators

Let us now assume that

(i) the noncrystallographic symmetry operators are close to true crystallographic symmetry, a situation known as pseudosymmetry (Zwart *et al.*, 2008 ▶); unlike ordinary NCS operators, which are local, pseudosymmetry operators are global and (within some tolerance; see Lebedev *et al.*, 2006 ▶) form a group with the crystallographic operators;
(ii) the twinning operators **T** *_k_* are a subset of the pseudosymmetry rotation operators **R**_*j*_.

Under these hypotheses, we have

In formulae (6), the notation {**T**|**t**} symbolizes the action of the operator defined by the rotation matrix **T** and the translational vector **t**, while {**S**|**s**}  {**T**|**t**} means the result of acting sequentially first with the operator defined by **T** and **t** and then with the operator defined by **S** and **s**. These equalities show that under the hypotheses stated above and for all choices of space-group symmetry operator *i*, pseudosymmetry operator *j* and twinning operator *k*, there exist operators labelled *i*′ and *j*′ that allow a simplification of the effect of a chosen twinning operator *k* on the symmetry copy *i* of the NCS copy *j*.

Thus, we can now rewrite (5),

Replacing this expression in (1) gives

where *I* ^noNCS^(**h**) = **F** ^noNCS^*(**h**)·**F** ^noNCS^(**h**) and *I*_1_(**h**) = **F**_1_*(**h**)·**F**_1_(**h**).

The terms within the summation over the twin index *k* in (8) describe the joint dependency of the observed intensity on the twin fractions and on the structural parameters. The part of the structure that does not follow the pseudosymmetry [ρ^noNCS^(**x**) in (2)] contributes to both terms within the summation. The part of the structure that does follow it, besides mixing with the noNCS part within the summation, also makes a contribution to the intensity that does not depend on the twin fractions (the first term on the right-hand side of equation 8). The larger the proportion of the structure following the pseudosymmetry, the smaller the dependency of the measured intensity on the twinning ratios. Notably, in the limit of no violations of the pseudosymmetry [*i.e.* **F** ^noNCS^(**h**) = 0] the diffracted intensity tends to the value computed for an untwinned crystal with pseudosymmetry.
